# Aberrant glycosylation associated with enzymes as cancer biomarkers

**DOI:** 10.1186/1559-0275-8-7

**Published:** 2011-06-03

**Authors:** Danni L Meany, Daniel W Chan

**Affiliations:** 1 Department of Pathology, Johns Hopkins University, Baltimore, MD 21231, USA

**Keywords:** Enzyme, Aberrant Glycosylation, Cancer Biomarkers, Glycosyltransferases, Glycoprotein, Glycan

## Abstract

**Background:**

One of the new roles for enzymes in personalized medicine builds on a rational approach to cancer biomarker discovery using enzyme-associated aberrant glycosylation. A hallmark of cancer, aberrant glycosylation is associated with differential expressions of enzymes such as glycosyltransferase and glycosidases. The aberrant expressions of the enzymes in turn cause cancer cells to produce glycoproteins with specific cancer-associated aberrations in glycan structures.

**Content:**

In this review we provide examples of cancer biomarker discovery using aberrant glycosylation in three areas. First, changes in glycosylation machinery such as glycosyltransferases/glycosidases could be used as cancer biomarkers. Second, most of the clinically useful cancer biomarkers are glycoproteins. Discovery of specific cancer-associated aberrations in glycan structures of these existing biomarkers could improve their cancer specificity, such as the discovery of AFP-L3, fucosylated glycoforms of AFP. Third, cancer-associated aberrations in glycan structures provide a compelling rationale for discovering new biomarkers using glycomic and glycoproteomic technologies.

**Summary:**

As a hallmark of cancer, aberrant glycosylation allows for the rational design of biomarker discovery efforts. But more important, we need to translate these biomarkers from discovery to clinical diagnostics using good strategies, such as the lessons learned from translating the biomarkers discovered using proteomic technologies to OVA 1, the first FDA-cleared In Vitro Diagnostic Multivariate Index Assay (IVDMIA). These lessons, providing important guidance in current efforts in biomarker discovery and translation, are applicable to the discovery of aberrant glycosylation associated with enzymes as cancer biomarkers as well.

## Introduction

Enzymes were one of the first protein molecules used as cancer biomarkers. Discovered in the early 1980s as a cancer biomarker for the early detection of prostate cancer, prostate specific antigen (PSA) is a serine protease[1]. With the exception of PSA, the increase in enzymatic activities or protein mass is not sensitive or specific enough for early detection of cancer[1]. Nevertheless, enzymes as cancer biomarkers have profound clinical utilities in the personalized approach to cancer diagnosis and treatment: Her-2/neu, a cell membrane surface-bound receptor tyrosine kinase, is a predictive marker to select breast cancer patients for treatment with trastuzumab (Herceptin)[2,3]. Urokinase plasminogen activator (uPA), a serine protease, is a prognostic marker for newly diagnosed breast cancer patients with lymph node-negative disease[4,4-7].

During the last decade, proteomic technologies have provided a new approach to identifying enzymes and related proteins as cancer biomarkers[8]. Glycoproteomic technologies that study glycans and glycoproteins are of particular interest in this regard because (1) aberrant glycosylation is a hallmark of cancer, reflecting cancer-specific changes in glycan biosynthesis pathways such as expression of glycosyltransferases and glycosidases[9-13] and (2) aberrant expression of these enzymes causes cancer cells to produce glycolipids and glycoproteins with modified glycans[12]. Advancements in glycoproteomic technologies have enabled comprehensive analyses of a given cell type or organism of all the glycan structures (glycomics) and of all the proteins containing glycans (glycoproteomics). Exploiting the difference in glycans between cancer and normal cells provides opportunities to discover new biomarkers for personalized cancer diagnosis and treatment. Discovery of these cancer-associated modifications of glycans on the glycoproteins may also improve on the specificity of existing cancer biomarkers. The feasibility of this approach has been demonstrated in the story of alpha-fetoprotein (AFP), a marker for hepatocellular carcinoma (HCC). AFP is not HCC-specific. Elevation of serum AFP levels also occurs in non-HCC conditions such as pregnancy, hepatitis, and liver cirrhosis[1]. In contrast, AFP-L3, consisting of core-fucosylated glycoforms of AFP, provides better specificity for HCC[14]. The improved cancer specificity of AFP-L3 is due to HCC's over-expression of enzyme fucosyltransferase Fut 8, which is required to produce core-fucosylated AFP and other enzymes pivotal for the synthesis of GDP-fucose, the substrate of the fucosyltransferase[15-18].

In this review, we provide examples of cancer biomarker discovery using aberrant glycosylation in three areas: (1) glycosyltransferases/glycosidases as cancer biomarkers, (2) improving on existing cancer biomarkers and, (3) discovery of new cancer biomarkers using glycomic and glycoproteomic approaches. We discuss the potential clinical applications of these biomarkers such as detection, prediction, and prognosis for a particular type of cancer. These types of clinical applications may be sufficient for a biomarker in the discovery phase; however, for a biomarker intended for clinical diagnosis, it would be better to define the clinical application at a specific decision-making point along the disease progression path[19].

### Glycosyltransferases/glycosidases as cancer biomarkers

Although multiple factors contribute to aberrant glycosylation in cancer--such as the availability and localization of nucleotide sugar donors and substrates--one of the primary mechanisms seems to be the differential expression of glycosyltransferases and glycosidases involved in the synthesis and catabolism of glycans. Therefore, these enzymes themselves may be used as cancer biomarkers (Table 1). The first example of such biomarkers is a family of enzymes that regulate the initial steps of mucin O-glycosylation: UDP-N-acetyl-D-galactosamine:polypeptide N-acetylgalactosaminyltransferases (ppGalNAc-T). Genome-wide association studies have shown that one single-nucleotide polymorphism (SNP) in GALNT1, the gene encoding a ppGalNAc-T enzyme, was statistically significant and inversely associated with the risk of ovarian cancer[20]. Furthermore, enzymes from this family of GALNT6 and GALNT14 were found to be elevated in breast and gastric carcinomas, which make them as potential tissue biomarkers[21-25]. Because aberrant glycoproteins as a result of these enzymes may be involved in promoting tumor invasion and metastasis, these enzymes could also be used as therapeutic targets. For example, screening for GALNT6 inhibitors would be valuable for development of novel therapeutic modalities against breast cancer, since over-expression of GALNT6 might contribute to mammary carcinogenesis [24].

**Table 1:** Enzymes associated with aberrant glycosylation as cancer biomarkers

Enzyme	Short form	Implication in aberrant glycosylation
polypeptide N-acetylgalactosaminyltransferase	ppGalNAc-T	Increased incomplete systhesis of O-glycans

N-acetylglucosamine transferase V	GlcNAcT-V	Increasedβ1-6 branching of N-glycans

α 2-3 sialyltransferases	ST3Gal I, ST3 Gal IV	Increased expression of sialylated glycans
	
α 2-6 sialyltransferase	ST6GalNAc	

Another glycosyltranferase that may be used as a cancer biomarker is UDP-N-acetyl-D-glucosamine: N-acetylglucosamine transferase V (GlcNAcT-V) that is responsible for β1-6 branching of N-glycans. Increased β1-6 branching of N-glycans as a result of over-expression of GlcNAcT-V in cancer plays an important role in tumor metastasis. Increased β1-6 branched N-glycans have been associated with lymph node metastasis in breast carcinoma[26]. Increased β1-6 branching of target proteins of GlcNAcT-V such as cadherin, integrin, and other cytokine receptors may enhance and promote tumor growth and metastasis[27-29]. Furthermore, Granovsky et al.[30] have shown that polyomavirus middle T antigen (PyMT)-induced tumor growth and metastasis were suppressed in adult mice lacking GlcNAcT-V. Thus, over-expression of GlcNAcT-V in cancer could be used as a biomarker of cancer progression and metastasis.

Sialyltransferases are another family of glycosyltranferases that are not only abnormally expressed in cancers[31-34], but are also implicated in carcinogenesis, progression, and metastasis. Over-expression of α 2-3 sialyltranferase I (ST3Gal-I) promotes mammary tumorigenesis in transgenic mice that over-express this enzyme under the control of the MUC1 promoter[35]. The over-expression of α 2-3 sialyltranferase III (ST3Gal-III) in pancreatic cancer cell lines indicates its roles in tumor progression[36]. Expression of α 2-6 sialyltransferase I (ST6GalNAc-I) in MDA-MB-231 breast cancer cells enhances the tumorigenicity of breast cancer cells[37]. Over-expression of sialyltransferases is generally associated with cancer progression and poor patient survival [22,38,39]. For example, over-expression of ST6GalNAc-II is related to poor patient survival in colorectal carcinomas as determined by reverse transcription PCR in 40 cases of colorectal carcinoma specimens and in "normal" mucosa of the same patients[40]. Interestingly, α 2-6 sialyltransferase I (ST6GalNAc-I) was associated with better prognosis in breast cancer in a study that compared mRNA levels of ST6GalNAc-I genes in 127 breast cancer tissues to 33 normal background tissues[22].

In addition to the role of tissue glycosyltransferases and glycosidases for risk assessment and prognosis, these enzymes may also be used as serum biomarkers for early detection of cancer and prediction of treatments. Ishizuka et al.[41] have shown that serial determination of serum α-L-fucosidase activity could be used for predicting the development of HCC in patients with liver cirrhosis--even before the detection of HCC by ultrasonography. Matsumoto et al. [42] demonstrated that plasma α-L-fucosidase activity was significantly correlated with progression-free survival in 24 breast cancer patients treated with trastuzumab monotherapy. Such activity indicates that it could be a predictive biomarker of sensitivity to trastuzumab treatment of breast cancer patients.

### Improving on Existing Cancer Biomarkers

Most of the U.S. Food and Drug Administration (FDA)-approved cancer biomarkers are glycoproteins. Discovery of the glycoforms related to cancer may help improve on these cancer biomarkers. Such endeavors require knowledge of whether carbohydrates on these proteins contribute to their use as cancer biomarkers. Since these biomarkers are measured by immunoassays, it would be important to understand whether antibodies used in these diagnostic tests are carbohydrate dependent. Based on the antibodies, clinically used cancer biomarkers can be divided into two groups: (1) carbohydrate-independent such as PSA, and (2) carbohydrate-dependent. Carbohydrate-dependent biomarkers can be further divided into two groups: (1) protein biomarkers such as CA 15-3/CA 27.29 and CA125, and (2) glycan markers such as CA 19-9. We discuss current research and potential ways to improve on biomarkers using PSA, CA 15-3/CA 27.29 and CA125, and CA 19-9 as examples.

### PSA

PSA is a proteolytic enzyme synthesized almost exclusively by the prostate. Under normal physiological conditions PSA concentrations in blood are low. However, under pathological conditions associated with the prostate--prostate cancer, prostitis, benign prostatic hyperplasia (BPH), and prostatic intraepithelial neoplasia--PSA concentrations in blood become elevated. Although elevation of serum PSA has been used clinically as a biomarker to help detect prostate cancer, it is not prostate cancer specific. The molecular isoforms of PSA such as free PSA and [-2] proenzyme PSA show moderate improvement in cancer specificity over PSA[1,43].

The antibodies currently used for measurement of PSA in diagnostic tests are carbohydrate-independent. As a result, current immunoassays for PSA are carbohydrate independent. However, PSA is a glycoprotein and has different glycoforms. Measurement of cancer-associated glycoforms of PSA may help improve the cancer specificity of PSA. Peracaula et al. [44] initially demonstrated this possibility--that altered glycosylation patterns allow the distinction of PSA from seminal fluid (normal) and prostate cancer LNCaP cells (tumor origins). Then, several glycoforms of PSA in serum to distinguish patients with prostate cancer from those with BPH were discovered: differential binding of PSA in serum to *Maackia amurensis *(MAA), a lectin that recognizes terminal α2-3 sialylation, by Ohyama et al.[45], and alpha1,2-fucosylated and beta-N-acetylgalactosaminylated PSA, by Fukushima et al.[46]. Furthermore, Meany et al. reported that *Sambucus nigra *(SNA)-bound PSA may improve on the percent free PSA in the diagnostic gray zone of percent free PSA between 10% and 20% in a subset of 21 patients (11 cancer and 10 non-cancer) and in a separate study of 16 additional subjects (8 cancer and 8 non-cancer)[47].

Aberrant glycoforms of PSA may help detect aggressive prostate cancers. Currently, aggressive and non-aggressive prostate cancers may all be initially diagnosed as clinically localized prostate cancers[48]. However, not all clinically localized prostate cancers are alike. Some are non-aggressive; even if left untreated, they neither reduce quality of life nor progress to cause mortality. Some are aggressive and at higher risk for recurrence after treatment or death from prostate cancer. Current methodologies (e.g., PSA, biopsy, and tumor staging) cannot accurately differentiate patients with aggressive or non-aggressive cancers. Thus we urgently need new biomarkers that accurately make this distinction at initial diagnosis of prostate cancer to allow each subgroup to receive the most appropriate therapy. In testing the hypothesis that aberrant glycoforms of PSA may help detect aggressive prostate cancers, we studied 13 individual glycan profiles of PSA enriched from prostate tissue specimens: 2 normal (N), 3 normal tissues from prostates with non-aggressive tumors (NAN), 3 cancerous tissues from the same prostates as NAN (NAT), 3 normal tissues from prostates with aggressive tumors (AN), and 2 cancerous tissues from the same prostates as AN (AT). Lectins *Maclura pomifera *(MPA) and *Ulex europaeus *(UEA) showed a trend of increased binding to PSA enriched from prostate tissue with increasing tumor aggressiveness. UEA was able to distinguish normal PSA in prostates with non-aggressive tumors from aggressive tumors (NAN vs. AN, p < 0.05). Therefore, aberrant glycoforms of PSA may help detect aggressive forms of prostate cancer.

### CA 15-3/CA 27.29 and CA125

Polymorphic epithelial mucin (MUC 1) is a highly O-glycosylated transmembrane glycoprotein, produced on the apical surfaces of the lining of hollow organs and glands toward the lumen by mucosal epithelial cells. Malignant transformation of mucosal epithelia causes MUC 1 to enter the bloodstream aberrantly. Measurement of MUC 1 in blood, therefore, serves as a guide for detecting and monitoring cancer. CA 15-3 and CA 27.29 are FDA-approved assays for the measurement of circulating MUC 1 antigen as an aid in monitoring disease recurrence or response to therapy in patients previously diagnosed with breast cancer. The most clinical utility of CA 15-3/CA 27.29 is in the setting of monitoring therapy in patients with advanced breast cancer through serial determinations of CA 15-3/CA 27.29 in conjunction with diagnostic imaging, history, and physical exams. Although high preoperative levels of CA 15-3/CA 27.29 are associated with adverse patient outcomes, CA 15-3/CA 27.29 has not been recommended by the National Academy of Clinical Biochemistry (NACB) or the American Society of Clinical Oncology (ASCO) for management of early stages of breast cancer, nor has it been recommended for detecting recurrence after primary breast cancer therapy--despite the fact that serial determination of CA 15-3/CA 27.29 levels after primary or adjuvant therapy can predict recurrence an average of 5-6 months before other symptoms or tests[49]. Finally, CA 15-3/CA 27.29 should not be used for early detection of breast cancer, due to a lack of sensitivity and specificity.

As a tumor marker for ovarian cancer, CA 125 is a MUC16 glycoprotein comprised of a carboxyl terminus anchor region, a dominant repeat region, and a predominantly O-glycosylated region. First described by Bast et al.[50] in 1981, CA125 is defined by the mouse monoclonal antibody OC 125, which recognizes the surface of ovarian tumor cells. Anchored to the epithelium by a transmembrane domain, CA125 is released to the extracellular space by enzymatic cleavage. Measurement of CA125 in blood, therefore, serves as an aid in monitoring disease recurrence or response to therapy in patients previously diagnosed with ovarian cancer. Despite the fact that CA125 alone lacks the sensitivity and specificity needed for screening asymptomatic women, it remains the single-best biomarker available for ovarian cancer[51-53].

Discovery of cancer-associated glycoforms of circulating MUC 1 and MUC 16 antigens may help improve their specificity for breast and ovarian cancers, respectively. Storr et al.[54] analyzed the O-glycans of MUC1 from the serum of a breast cancer patient and found them to be comprised mainly of sialylated core 1 type glycans. Jankovic et al.[55] compared glycans of CA-125 (MUC16) isolated from amniotic fluid to the CA-125 from an OVCAR3 ovarian cancer cell line. They found a significant increase in the reactivity of OVCAR3 CA-125 with the lectin E-PHA compared with CA-125 from amniotic fluid. Once the cancer-associated glycan structure is identified, antibodies that specifically target the structure may be developed for improving on the current diagnostic tests.

### CA 19-9

Cancer progression is often associated with changes in the glycan structures of glycolipids. The glycosyltransferases and glycosidases that act on glycoproteins also act on glycolipids, resulting in aberrant cancer-associated glycan structures shared by glycoproteins and glycolipids. Indeed, once the aberrant cancer-associated glycans in glycolipids are identified, monoclonal antibodies that target the aberrant glycans may be relatively easy to produce for several reasons: (1) their structures can be elucidated, (2) glycolipids can be purified to homogeneity, and (3) a purified glycolipid maintains antigenicity. For these reasons, aberrant glycans on glycolipids have been extensively studied by the monoclonal antibody approach[56]. In fact, many monoclonal antibodies directed to human tumor cells or to tissues that show a distinctive reactivity to the specific type of human cancer have been identified as being directed to glycolipids, such as N-19-9 antibody for sialyl Lewis^a ^associated with gastrointestinal/pancreatic cancers[56,57].

N-19-9 antibody is used in CA 19-9 assay to detect aberrant sialyl Lewis^a ^glycan. Because this aberrant glycan is predominantly expressed on mucins in serum from patients with GI malignancies, it has been used as a cancer biomarker for patients with GI malignancies. One caveat of using CA 19-9 is that patients known to be genotypically negative for the Lewis^a ^blood group antigen will not produce the CA 19-9 antigen--even in the presence of malignant tissue[58]. Another problem with CA 19-9 is that sialyl Lewis^a ^glycan is neither a specific product of a specific tumor nor a tumor only[56]. This problem may be alleviated by using glycoproteomic approaches to identify glycoproteins accompanying sialyl Lewis^a ^glycan whose differential expressions are also associated with a specific type of cancer[59].

### Discovery of Cancer Biomarkers Using Glycomic and Glycoproteomic Approaches

Technological advancements in the field of Glycobiology have allowed comprehensive comparisons of glycans and glycoproteins between normal and tumor cells to identify differential expression of these cancer-associated glycans and glycoproteins as potential cancer biomarkers. The glycomic approach uses various methods to release glycans off glycoproteins or glycolipids and to analyze only the glycans, whereas the glycoproteomic approach separates glycoproteins or fractions of glycoproteins using affinity or other enrichment methods and analyzes the proteins after they are released from the glycans. Exploiting the difference in glycans and glycoproteins between cancer and normal cells using glycomic and glycoproteomic approaches provides an opportunity to discover new biomarkers for personalized cancer diagnosis and treatment.

### Cancer Biomarkers Discovered by Glycomic Approaches

Glycomic studies have been carried out to identify changes in serum glycan profiles. Kyselova et al. [60] compared serum glycan profiles of 10 healthy men and 24 men with confirmed prostate cancer who were undergoing androgen-deprivation therapy (ADT) due to cancer metastasis. The sera for these cancer patients were obtained at the time of starting ADT. This study identified 12 glycan structures that significantly differentiated between cancerous and normal sera (2 glycans decreased in cancer and 10 increased in cancer) with 6 of the glycans fucosylated and 9 of the glycans sialylated to a different degree (mono-, di-, and trisialylated structures). Using the same method in a different study, Kyselova et al. [61] compared the serum glycomic profiles of 27 non-breast cancer women and 82 breast cancer patients in various stages (12 in stage I, 11 in stage II, 9 in stage III, and 50 in stage IV). Results from this study, including a heterogeneous population of patients that resembled a true breast cancer population, showed results similar to the prostate cancer study--that breast cancer progression appeared to be associated with increased sialylation and fucosylation of glycans in sera.

Changes in serum glycan profiles--demonstrated in prostate, breast, and other types of cancer--have also been exploited as cancer biomarkers in liver cancer for two reasons: First, the vast majority of glycoproteins in serum are produced by hepatocytes; second, the asialoglycoprotein receptors and mannose/N-acetylglucosamine (GlcNAc) receptors in the liver have important roles in the clearance of aberrantly glycosylated proteins[62]. Thus, changes in the serum N-glycome profile may reflect pathological changes in the liver.

Multiple non-invasive tests based on serum protein glycomics have been developed for monitoring liver fibrosis (GlycoFibro test), detecting liver cirrhosis (GlycoCirrho test), and for screening HCC (GlycoHCC test). All these tests use a DNA sequence analyzer to generate profiles of the serum protein N-glycans of liver disease patients. The GlycoFibro test calculates the log ratio between the agalacto glycans (NGA2FB) and the fully galactosylated triantennary glycans (NA3), which appear to rise gradually with an increasing fibrosis stage[63]. The GlycoCirrho test can distinguish compensated (early stage) cirrhosis from non-cirrhotic chronic liver disease with 79% sensitivity and 86% specificity[62]. The GlycoHCC test, using the log ratio of a branch alpha (1,3)-fucosylated triantennary glycan (elevated in HBV patients with cirrhosis) to a bisecting core alpha(1,6)-fucosylated biantennary glycan (elevated in HBV patients with HCC), shows similar sensitivity and specificity to that of AFP in screening HCC from patients with cirrhosis[64].

Besides serum, glycomic analysis has been applied to cells from culture and tissue origins. Goetz et al.[65] showed increased fucosylation in O-glycans isolated from invasive cancer cells that could potentially be considered as a measure of breast cancer invasiveness and tumor progression. Lattova et al.[66] identified glycan changes in human breast carcinoma cell lines after treatment with Herceptin and Herceptin/Lipoplex, which helped study the role of glycosylation during antibody treatment. A glycomic approach has also been applied to glycolipids. Using normal colorectal epithelial cells and colorectal cancer cells that were highly purified with the epithelial cell marker CD326, Misonou et al. [67] identified three specific alterations in glycosphingolipids in cancer cells compared to normal: increased ratios of type-2 oligosaccharides, increased α 2-3, and α 2-6 sialylation, and increased α1-2 fucosylation. Specifically, a shift from type-1 dominant normal colorectal epithelial cells to type-2 dominant colorectal cancer cells was found in five patients with hepatic metastasis. These cancer-associated glycan structures may be used to discover glycoproteins as cancer biomarkers for patients with more aggressive cancers and for follow-up of cancer progression using the glycoproteomic approaches.

### Cancer Biomarkers Discovered by Glycoproteomic Approaches

Glycomic analysis provides direct and structural information of aberrant glycans associated with cancer. Such information may also be provided indirectly by studies of the expression of glycosyltransferases, enzymes involved in the synthesis of glycans. Information provided by both direct and indirect approaches has been used in glycoproteomics to identify proteins with aberrant glycosylation in tissue, serum, and cultured cells as potential cancer biomarkers.

To identify biomarkers for ovarian cancer, Abbott et al.[68] first studied the expression of glycosyltransferases in endometrioid ovarian tumor tissues using quantitative real-time PCR and identified increased transcripts of enzymes responsible for core fucosylation (FUT 8) and bisecting glycans (GnT-III). Assuming that these two glycosylation changes may be related to endometrioid ovarian cancer, Abbott et al. [69] used lectins AAL and E-PHA to enrich the glycoproteins with core fucosylation and bisecting GlcNAc, respectively, from endometrioid ovarian cancer and non-diseased human ovary tissues, and identified several glycoproteins with these glycosylation changes that have higher abundance in cancer than in normal tissues. Among these glycoproteins, periostin and thrombospondin were validated in tissue and serum using lectin blots, although only a few samples were used in the validation[69]. Applying a similar approach to matched normal (non-diseased) and malignant tissue isolated from patients with invasive ductal breast carcinoma, this group enriched glycoproteins with increased beta(1,6)-branched N-linked glycans using L-PHA that does not bind to non-diseased breast epithelial cells, but binds to cells progressed to invasive carcinoma. This study identified 12 proteins that increased in all 4 matched tumor cases relative to normal tissues[70], including periostin and haptoglobin-related protein precursor or haptoglobin.

In serum the most successful story of applying an aberrant glycosylation approach to discover cancer biomarkers is the use of increased fucosylation for HCC. Using woodchucks as the animal model--whose HCC resemble that of human HCC--Block et al. [71] identified that woodchucks diagnosed with HCC have dramatically higher levels of serum-associated core α 1,6-linked fucose compared to woodchucks without HCC. Extending this finding, this group used 2D-gel proteomics to identify glycoproteins with altered core fucosylation. One such glycoprotein--Golgi Protein 73 (GP73)--not only elevated and hyperfucosylated in animals with HCC, but also in the serum of humans with the diagnosis of HCC[72]. Applying a similar 2D-gel proteomic strategy to human sera, Comunale et al.[73] identified 19 serum proteins to be hyperfucosylated in HCC. Among these 19 proteins, fucosylated hemopenxin and fetuin A were confirmed in another study by the same group in a cohort of 300 serum samples using lectin-based high-throughput plate-based assays were confirmed to have an ROC area under the curve (AUC) of 0.95 and 0.87, respectively, in the differentiation of HCC from non-HCC conditions [74]. A separate study from the same group showed that the combination of fucosylated kininogen, AFP, and GP 73 gave an optimal sensitivity of 95% at a specificity of 70% and an AUC of 0.94 for identifying patients with HCC[75]. Nevertheless, the clinical utilities of GP73 are still controversial: Mao et al. [76] showed that GP73 was an accurate serum marker for detection of HCC and recurrence after surgery, whereas Yamamoto et al. and Ozkan et al.[77,78] showed that it was not useful in the diagnosis of HCC, in monitoring treatment response, or in prognosis.

Serum acute-phase reactants were often identified by glycoproteomic approaches to be the carriers of aberrant glycans associated with cancer. For example, Abd Hamid et al. [79] demonstrated acute-phase proteins α1-acid glycoprotein, α1-antichymotrypsin, and the haptoglobin β-chain to be contributors to a 2-fold increase in the monogalactosylated triantennary glycan structure containing alpha1,3-linked fucose in breast cancer patients as compared to the controls. Although these serum acute-phase reactants may be markers of cancer, they are not specific for a type of cancer, nor are the aberrant glycosylation of these serum acute-phase reactants. For example, serum concentration of fucosylated haptoglobin has been shown to be increased in prostate, colon, breast, ovarian, and liver cancers[70,80-83]. Nevertheless, a multiple-marker strategy combining markers with independent clinical values may still benefit from these markers that increase the sensitivity but not the specificity for cancer detection[84].

It is well-known that aberrant O-glycans provoke immune responses[85-88]. Serum autoantibodies against those glycans, therefore, have become another promising area for discovery of cancer biomarkers. This was made especially feasible by development of the chemoenzymatic synthesis of O-glycopeptides. Using microarrays deposited with the synthesized O-glycopeptides, Wandall et al. [89] screened for autoantibodies generated to aberrant glycoforms of MUC1 as cancer biomarkers for early detection of breast, ovarian, and prostate cancers. Similarly, Wang et al.[90] used glycan microarrays comprised of Globo H, a cancer-associated carbohydrate antigen highly expressed on breast cancer cells and other related structures, for quantitative analysis of their respective autoantibodies present in the plasma of breast cancer patients and normal blood donors. This study showed that the amount of autoantibodies against Globo H from breast cancer patients were significantly higher than normal blood donors, providing a new tool for possible breast cancer diagnosis[90].

Besides tissue and serum, cell lines offer good models to discover cancer biomarkers. Dai et al. [91] identified aberrantly α1,6-fucosylated glycoproteins related to hepatocellular carcinoma (HCC) metastasis in MHCC97-H and MHCC97-L cells with high and low metastatic potentials. This study implied that the alteration of CK8, annexin I, and annexin II both in their expression levels and in their glycan parts might be related to metastatic ability, and may also play a critical role in the process of HCC metastasis. HT-29 human colon epithelial cancer cells are a cellular model of colon cancer progression, as they can either proliferate or differentiate into enterocyte phenotype. Vercoutter-Edouart et al. [92] identified membrane-bound N-glycoproteins from HT-29 cells and significant changes in a 2,3- and a 2,6-sialylation of these membrane glycoproteins contributed by solute carrier family and adhesion proteins.

A glycoproteomic approach has also been used to identify predictive cancer biomarkers. Although the majority of advanced ovarian carcinomas initially respond to chemotherapy, a significant portion of them fail to respond successfully to further treatments. Chemoresistance thus represents a major obstacle in attempts to improve clinical outcome. The high prevalence and poor prognosis of ovarian cancer emphasize the need to identify prognostic markers that can be used to select patients to receive new, individualized therapies. One cause of chemoresistance in human cancer may be the elevated expression or activity of ATP-binding cassette transporters. Glycoproteomic analysis of paclitaxel-sensitive and resistant human epithelial ovarian cancer cell lines identified putative biomarkers that were remarkably upregulated in resistant cell lines and may represent biomarkers for paclitaxel resistance in ovarian cancer[93].

### Cancer Biomarkers Discovered by Functional Glycoproteomic Approaches

Glycoproteins with aberrant glycosylation that are differentially expressed in normal and cancer cells--for which we have given a few examples in the previous section--may be used as cancer biomarkers. But functionally it is unknown how these proteins are responsible for cancer progression. A functional glycoproteomic approach could help answer this question by (1) identifying target proteins of these glycosyltransferase genes implicated in cancer, using the glycosyltransferase gene knockout or knockdown cell models and (2) identifying the functional roles of these aberrant glycosylated proteins in cancer cell invasion and metastasis in vivo and in vitro. Glycoproteins with aberrant glycosylation that are functionally responsible for cancer progression are more likely to be used as cancer biomarkers.

Examples of glycoproteins with aberrant glycans implicated in cancer progression include tissue inhibitors of metalloproteinase-1, identified as target proteins of N-acetylglucosamine transferase V (GlcNAcT-V). The tissue inhibitor of metalloproteinase-1 (TIMP-1) is an endogenous inhibitor of matrix metalloproteinase that plays a critical role in invasion, migration, and malignant transformation of cancer cells. The aberrant glycans on TIMP-1, induced by GlcNAcT-V, may affect the properties of binding with gelatinases, presumably by conferring a steric hindrance arising from the massiveness of glycosylation and an electrostatic repulsion arising from the attachment of acidic residues to the binding to gelatinases[94,95]. Because of the functional role of aberrant glycosylation of TIMP-1, Ahn et al. [96] generated antibodies recognizing an aberrant glycoform of TIMP-1. Such efforts will be very helpful in the development of immune-based assays to evaluate the clinical performance and utilities of aberrant glycoforms of TIMP-1 in a personalized approach to cancer diagnosis and treatment.

## Conclusions

Cancer biomarkers could be the driving force in the personalized approach to cancer diagnosis and treatment. As a hallmark of cancer, aberrant glycosylation allows for the rational design of biomarker discovery research. First, changes in glycosylation machinery such as glycosyltransferases/glycosidases could be used as cancer biomarkers. Second, differential expression of these enzymes in the compared cancer cells may point to specific cancer-associated aberrations in glycan structures. In the case of AFP-L3, the increased fucosylation of AFP was attributed to the increased expression of α1-6 fucosyltransferase in hepatoma tissues[97]. Therefore, cancer-associated aberrations in glycan structures can be used to improve on existing cancer biomarkers, most of which are glycoprotein. Furthermore, cancer-associated aberrations in glycan structures provide a compelling rationale for the discovery of new biomarkers through the isolation and identification of glycoproteins that contain these glycan structures.

There is no doubt that we need to develop glycomic and glycoproteomic technologies with higher throughput and better sensitivity, specificity, and reproducibility. More important, we need to translate these biomarkers into clinical diagnostics[19]. Subjecting them through a vigorous validation process using high-quality, well-annotated clinical specimens is necessary. And availability of large serum banks that could provide such specimens, therefore, will help facilitate the translation of biomarkers from discovery into clinical diagnostics.

## Competing interests

The author declares that they have no competing interests.

## Authors' contributions

DLM reviewed the literature and drafted the manuscript. DWC participated in its design and discussion. All authors read and approved the final manuscript.
